# Bacterial and diazotrophic diversities of endophytes in *Dendrobium catenatum* determined through barcoded pyrosequencing

**DOI:** 10.1371/journal.pone.0184717

**Published:** 2017-09-20

**Authors:** Ou Li, Rong Xiao, Lihua Sun, Chenglin Guan, Dedong Kong, Xiufang Hu

**Affiliations:** 1 College of Life Science, Zhejiang Sci-Tech University, Xiasha, Hangzhou, PR China; 2 Zhejiang Academy of Medical Sciences, Hangzhou, PR China; 3 Agricultural Experiment Station, Zhejiang Univesity, Hangzhou, PR China; The National Orchid Conservation Center of China; The Orchid Conservation & Research Center of Shenzhen, CHINA

## Abstract

As an epiphyte orchid, *Dendrobium catenatum* relies on microorganisms for requisite nutrients. Metagenome pyrosequencing based on 16S rRNA and *nif*H genes was used to characterize the bacterial and diazotrophic communities associated with *D*. *catenatum* collected from 5 districts in China. Based on Meta-16S rRNA sequencing, 22 bacterial phyla and 699 genera were identified, distributed as 125 genera from 8 phyla and 319 genera from 10 phyla shared by all the planting bases and all the tissues, respectively. The predominant *Proteobacteria* varied from 71.81% (GZ) to 96.08% (YN), and *Delftia* (10.39–38.42%), *Burkholderia* (2.71–15.98%), *Escherichia/Shigella* (4.90–25.12%), *Pseudomonas* (2.68–30.72%) and *Sphingomonas* (1.83–2.05%) dominated in four planting bases. *Pseudomonas* (17.94–22.06%), *Escherichia/Shigella* (6.59–11.59%), *Delftia* (9.65–22.14%) and *Burkholderia* (3.12–11.05%) dominated in all the tissues. According to Meta-*nif*H sequencing, 4 phyla and 45 genera were identified, while 17 genera and 24 genera from 4 phyla were shared by all the planting bases and all the tissues, respectively. *Burkholderia* and *Bradyrhizobium* were the most popular in the planting bases, followed by *Methylovirgula* and *Mesorhizobium*. *Mesorhizobium* was the most popular in different tissues, followed by *Beijerinckia*, *Xanthobacter*, and *Burkholderia*. Among the genera, 39 were completely overlapped with the results based on the 16S rRNA gene. In conclusion, abundant bacteria and diazotrophs were identified in common in different tissues of *D*. *catenatum* from five planting bases, which might play a great role in the supply of nutrients such as nitrogen. The exact abundance of phylum and genus on the different tissues from different planting bases need deeper sequencing with more samples.

## Introduction

*Dendrobium catenatum* Lindl. is one of the most popular epiphytic medicinal orchids in South Asia and Southeast Asia [1]. Stems of *D*. *catenatum* contain rich polysaccharide and dendrobine contents, which makes them valuable for relieving stomach upsets, promoting body fluid production, nourishing ‘‘yin” and antipyresis [2], relieving throat inflammation and fatigue, reducing peripheral vascular obstruction, and enhancing immunity. Therefore, *D*. *catenatum* has been listed in the 2010 edition of the *Chinese Pharmacopoeia*, under the name ‘Tiepishihu’ as the sole origin of the herb [3]. The many desirable functions have greatly increased the demand for Tiepishihu, thereby resulting in severe depletion of the wild resources of this plant. Therefore, in order to meet the increasing demand, artificial cultivation has become the main source of *D*. *catenatum*.

It is well known that fungal endophytes have profound effects on plant ecology, fitness, evolution, and even the diversity and structure of plant communities [4]. As epiphytic or lithophytic plants with minute seeds [5], *D*. *catenatum* plants vitally require fungi as their main source of essential nutrients for growth and secondary metabolism [6]. Knowledge of the diversity and functions of endophytic fungi in dendrobium has been accumulating. However, the so-called mycorrhiza helper bacteria (MHB) or “satellite” bacteria were found to be commonly occurring in ectomycorrhiza and in arbuscular mycorriza associations [7] since Bowen and Theodorou first reported the promotion of *Rhizopogon luteolus* on *Pinus radiate* roots [8]. The popular MHB are closely associated with mycorrhizal fungi (MF) with taxonomically diverse bacterial groups [9], and the majority of bacteria have a stimulating effect on the mycelial growth and mycorrhiza formation [10]. This indicates that not only single species, but entire microbial communities, may have evolved to live in close association with mycorrhizal fungi and plants.

Plant associated bacteria are commonly recognized to have a great and often favorable impact on plant growth and development, due to nitrogen fixation, production of plant growth regulators, improvement of water uptake and mineral nutrition, and biosynthesis of fungicidal and/or bactericidal substances, thus reducing the number of phytopathogens [11]. For epiphyte or lithophyte *D*. *catenatum*, nitrogen-fixation is one of the most important functions provided by the unique ability of diazotrophic bacteria. Therefore, it is worth learning about the diversity of diazotrophic bacteria in the plants of *D*. *catenatum*.

However, not much is known about the composition and functional activity of orchid-associated bacteria, let alone diazotrophic bacteria in particular. Some studies on microbial diversity, localization and functional activity of orchid-associated bacteria in several greenhouse and wild-grown terrestrial and epiphytic orchids revealed an abundance of heterotrophic and phototrophic bacteria on the roots of some cultivated tropical orchids of *Calanthe*, *Acampe* and *Dendrobium* genera. As observed through scanning electron microscopy, microbial clusters can occupy the surface and inner root tissues, particularly the velamen, and be submerged within the intercellular matrix. The multilayered structure of the velamen, which may protect the associated bacteria from various biotic and abiotic factors, represents a suitable econiche for colonization by cyanobacteria. The functional role of the isolated strains was proven by high nitrogen-fixing activity of the orchid-associated cyanobacteria, and indole-3-acetic acid (IAA) production by heterotrophic bacteria. The intimate relations of cyanobacteria with their hosts create a unique symbiotic consortium, which guarantees an ecological stability and nutrient supply for both partners [12]. The MHB, with cyanobacteria being a well-studied example, have become the focus of recent interest. Thus, the diversity and roles of other orchid diazotrophic endophytes remain largely unexplored.

High-throughput sequencing is a promising method for investigating microbial community structure and diversity, as it provides enough sequencing depth to cover complex microbial communities [13]. Thus far, it has been applied to analyze microbial communities in both environment [14] and host [15]. However, few studies have been conducted using this method to investigate *D*. *catenatum*. Both phylogenetic (e.g., 16S rRNA, *gyr*B, *rec*A, and ribosomal intergenic regions) and functional marker genes (e.g., *amo*A, *nir*S, *nir*K, *nif*H, *dsr*AB, and other biogeochemically important genes) are very useful for studying phylogenetic relationships among different organisms, for analyzing microbial community structure, and/or for monitoring the physiological status and functional activities of microbial populations in natural environments [16].

The purpose of this study is to assess the diversity of bacterial and diazotrophic endophytes in *D*. *catenatum* based on metagenome targeting of 16S rRNA and *nif*H genes. Thess results of this study may also be beneficial for the study of the functions and interactions of bacteria with their hosts. To the best of our knowledge, this study is the first application of PCR-based Illumina Miseq pyrosequencing for the characterization and comparison of multiple *D*. *catenatum* samples.

## Materials and methods

### *D*. *catenatum* plant samples

*D*. *catenatum* plants were randomly collected from planting bases in Zhejiang (ZJ), Fujian (FJ), Yunnan (YN), Guizhou (GZ) and Guangxi (GX) provinces in China (shown in Table 1). All the planting bases belong to private land, and the owner, one of the co-auther, gave permission to conduct the study on these sites. From each site, five sub-samples were collected and mixed to provide a unique representative sample for endophytic diversity analysis. All the plants (2 years old) exhibited a healthy appearance and were used for total DNA extraction.

**Table 1:** Location of five geographically distributed planting bases of *D*. *catenatum*.

Sample	Province	City	Latitude	Longitude
**ZJ**	Zhejiang	Jinhua	28°90’ N	120°03’ E
**GZ**	Guizhou	Guiyang	26°35’ N	104°50’ E
**FJ**	Fujian	Quanzhou	24°54’ N	118°37’ E
**YN**	Yunnan	Mangshi	24°22’ N	98°31’ E
**GX**	Guangxi	Rongxian	22°36’ N	110°47’ E

### DNA extraction

The *D*. *catenatum* plants were washed under running tap water to remove adhering soil particles and the majority of microbial epiphytes. Root, stem and leaf tissues were separated and sterilized to eliminate remaining microorganisms using a series of treatments consisting of sterile distilled water, 70% ethanol, and sodium hypochlorite solution (2.5% available Cl^-^). Total genomic DNA was extracted from about 0.2 g fresh plant tissues using the modified cetyltrimethyl ammonium bromide (CTAB) method [17], with the incubating time at 65°C prolonged to 90 min. The quality of the extracted DNA was analyzed using the Nanodrop method. The DNA was stored at −20°C.

### Amplicon library construction

The DNA concentration in each sample was measured using a NanoDrop ND-1000 spectrophotometer (Nanodrop Technologies, Wilmington, DE, USA) to verify that samples had concentrations in excess of 50 ng/ul, which is the value required for metagenomic analyses. The fragments of 16S rRNA and *nif*H genes were amplified using the metagenomic DNA and Super-Therm Taq DNA polymerase (JMR, UK). Primer pair fM1/rC5 was used for amplification of the 16S rRNA gene [18], and PolF/PolR and PolF/ZehrR pairs were used for amplification of the *nif*H gene [19, 20]. The 5’-fused primer includes an inserted 7 nucleotide ‘barcode’; the barcode is permuted for each sample and allows the identification of individual samples in a mixture in a single pyrosequencing run [21].

Based on the manufacturer’s instructions, the PCR reaction mixture (50 μL) contained 1 μL Taq polymerase (2.5 unit), 3 μL primer set, 1BF-2BR (10 pmol), 5 μL of reaction buffer, 15 mM MgCl_2_, 5 μL of 2 mM dNTP, 5 μL template DNA, and 28 μL sterilized water. Thirty cycles, with denaturation at 94°C for 60 s, annealing at 54°C for 40 s, and extension at 72°C for 45 s, were followed by a final incubation at 72°C for 8 min. After PCR analysis on agarose gel (1%), the specific bands were purified using a PCR purification kit (Sangon Biotech, Shanghai, China). The barcode-tagged fragments were quantified using a Qubit^®^ dsDNA HS Assay kit with a Qubit2.0 fluorometer (Life Technologies, Grand Island, NY, USA) and pooled in approximately equal concentrations to ensure equal representation of each sample. These samples were sequenced using 2×300 paired-end (PE) Illumina Miseq at the Shanghai Sangon Biotech Co. Ltd (Shanghai, China).

### Illumina sequencing and analysis

PCR products with unique indices from each library were taken in equal nanogram quantities and subjected to 200-nucleotide paired-end multiplex sequencing using an Illumina GAIIx sequencer. Image analysis and base calling were performed using Illumina Analysis pipeline (Version 2.2).

After sequencing, FLASH v1.2.7 was used to separate the PE reads from each sample according to their barcode sequence, then overlapped to assemble the tag sequences. With that, the assembled sequence was completed with removing the sequences whose lengths did not meet the minimum quality filter criteria, and removing the barcode sequences and the joint sequences, thus allowing the acquisition of high quality and credibility clean data. The sequences selected above were defined as ‘raw reads’ for each sample. All raw sequence data had been deposited in the NCBI Sequence Read Archive database and the accession number is SRR3035220.

The raw reads were de-noised using pre.cluster’ (http://www.Mothur.org/wiki/Pre.cluster) to remove sequences that were likely due to sequencing errors [22]. Unique sequences were then aligned against SILVA [23] and chimeric sequences were removed using chimera.uchime [24]. After sequence quality control and filtration, the effective sequences of each sample were clustered using QIIME 1.8.0 to build operational taxonomic units (OTUs) with a cutoff value of 97% sequence identity. In order to analyze species composition more accurately, the RDP Classifier of the Ribosomal Database Project (RDP) [25] was used for all OTU representative sequences species classification analysis at a confidence threshold of 80%. Taxonomic assignment from phylum level to genus level was assigned based on the hits, with abundance graphs plotted based on the number of hits. Heatmaps were plotted using MG-RAST. The Venn diagram was constructed with the Venn diagram plotter jquery.venny, a tool developed by genotoul bioinfor (http://bioinfo.genotoul.fr/). Diversity indices were calculated using SPADE software [26]. The rarefaction curve and Alpha rarefaction calculations were performed using Mothur [27], including Chao1, ACE values, Shannon indices and coverage. For beta diversity analysis, NMDS plot based on RDP Classifier taxa was conducted using un-weighted UniFrac distance implemented in QIIME [28].

Meta-*nif*H sequence analysis was performed using the software package QIIME [29] version 1.8.0, according to the Qiime tutorial (http://qiime.org/) with some modified methods. Chimeric sequences were removed using usearch61 [30] with *denovo* models. Sequences were clustered into Operational Taxonomic Units (OTUs) at the thresholds of 97%, 95%, 92%, and 90% sequence similarities using uclust. Each OTU was then assigned to a taxonomic group using BLAST [31] (e-value: 1e-10) with the NCBI NT database (Excluding uncultured/environmental sample sequences). Rarefaction and rank abundance curves were calculated from OTU tables generated at 92% similarity using alpha diversity and rank abundance scripts within the QIIME pipeline. *β*-diversity analysis generated a NMDS plot using un-weighted UniFrac distance implemented in QIIME. Analysis of Variance (ANOVA) was carried out to test the difference between samples using STAMP.

## Results

Based on the metagenome sequencing and data analysis, 141,196 16S rRNA gene sequences and 633,624 *nif*H sequences were obtained from 15 *D*. *catenatum* samples, and the number of reads per sample ranged from 7,582 to 12,052 and 23,292 to 61,285 (S1 Table), respectively. GZL had the highest bacterial ratio and ZJS had the lowest bacterial ratio, while GZL had the highest *nif*H ratio and ZJL had the lowest *nif*H ratio.

**Fig 1:**
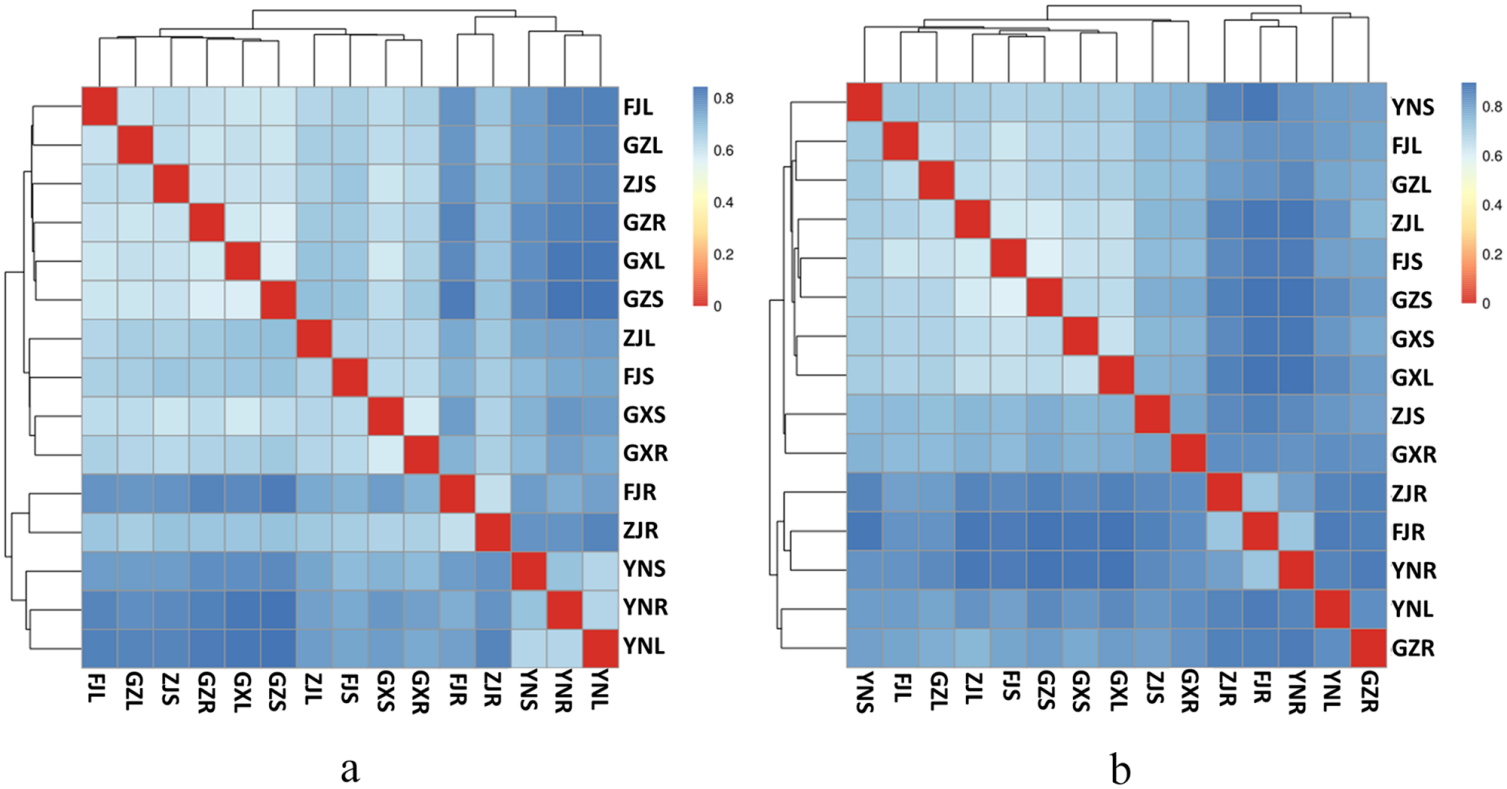
The unweighted heatmap based on 16S rRNA (a) and *nif*H (b) communities.

The relationships among the microbial communities of the different samples were investigated using unweighted heat map. In the resulting heat map, different distances were observed between the microbial communities of individual samples (Fig 1). In the case of the bacterial community (Fig 1b), stem and leave samples tended to cluster closer to each other, which was consistent with the results of the NMDS plot (S2 Fig). For the diazotrophic community (Fig 1b), stem and leaf samples appeared to cluster together, while the root samples exhibited another cluster. Therefore, some similarity existed between the bacterial and diazotrophic communities of the samples.

### Taxonomic diversity of bacterial endophytes

Rarefaction curves were generated to facilitate comparison of the sequencing effort among the samples (S1a Fig). None of the curves approached a plateau, suggesting that further sequencing would have resulted in more OTUs in each sample. As shown in Table 2, GZ and FJ samples displayed relatively higher species richness than the samples from the other three locations with regard to the number of OTUs, Shannon Wiener, Chao 1, and ACE indices. In total, the Shannon Wiener indices of the roots were higher than those of the stems and leaves with the exception of GX and GZ samples. For the planting bases, GZ showed the highest Shannon Wiener index, followed by FJ and GX, while YN had the lowest index. According to the heat map (Fig 1), the leaf samples tended to cluster together with stems, and roots samples tended to cluster in another distinct group.

**Table 2:** Diversity indices of the *D*. *catenatum* samples.

Sample_ID	Seq_num	OTU_num	Shannon_index	ACE_index	Chao1_index	Coverage
ZJR	9201	2809	6.489119	17212.28	9045.055	0.780459
ZJS	7582	1382	4.334109	4686.824	3010.457	0.885782
ZJL	8466	1117	3.629259	4235.37	2642.424	0.916135
FJR	9698	1637	5.420732	6494.123	3826.961	0.89668
FJS	8599	1415	4.84392	6659.969	3805.294	0.889987
FJL	9415	2115	5.042184	9422.082	5883.846	0.847584
GXR	9453	1149	4.356798	4479.672	2575.017	0.924786
GXS	9781	1295	4.320648	4760.654	2917.015	0.916777
GXL	9685	3023	6.120562	22514.13	10996.37	0.76634
YNR	8196	819	4.620535	3242.374	2049.062	0.940337
YNS	8673	643	3.654305	2174.8	1483.373	0.956878
YNL	8625	597	2.710412	2044.082	1495.162	0.95942
GZR	10252	2261	4.638355	12487.28	6573.458	0.843835
GZS	11518	2785	5.898415	13599.43	7904.747	0.835128
GZL	12052	2012	4.905339	9805.819	5872.156	0.886077

Across all the fifteen samples, a total of 22 bacterial phyla were identified, but only four had an average abundance of greater than 1% (S3 Fig), which accounted for 98.35% of the total effective bacterial sequences. The four dominant phyla were *Proteobacteria* (84.40%), *Bacteroidetes* (6.13%), *Firmicutes* (5.59%), and *Actinobacteria* (2.23%). The results showed that *Proteobacteria* was the predominant phylum, which was dominated by *Gammaproteobacteria* (42.53%). On the genus level, there were 699 genera identified from *D*. *catenatum*, 12 of which had over 1% reads. The predominance at the phylum and class levels was driven by the high abundance of five dominant genera with over 4.5% reads in average, such as: *Pseudomonas* (20.04%), *Delftia* (16.59%), *Escherichia/Shigella* (9.46%), *Burkholderia* (6.62%) and *Methylobacterium* (4.52%).

#### Diversity between planting bases

**Fig 2:**
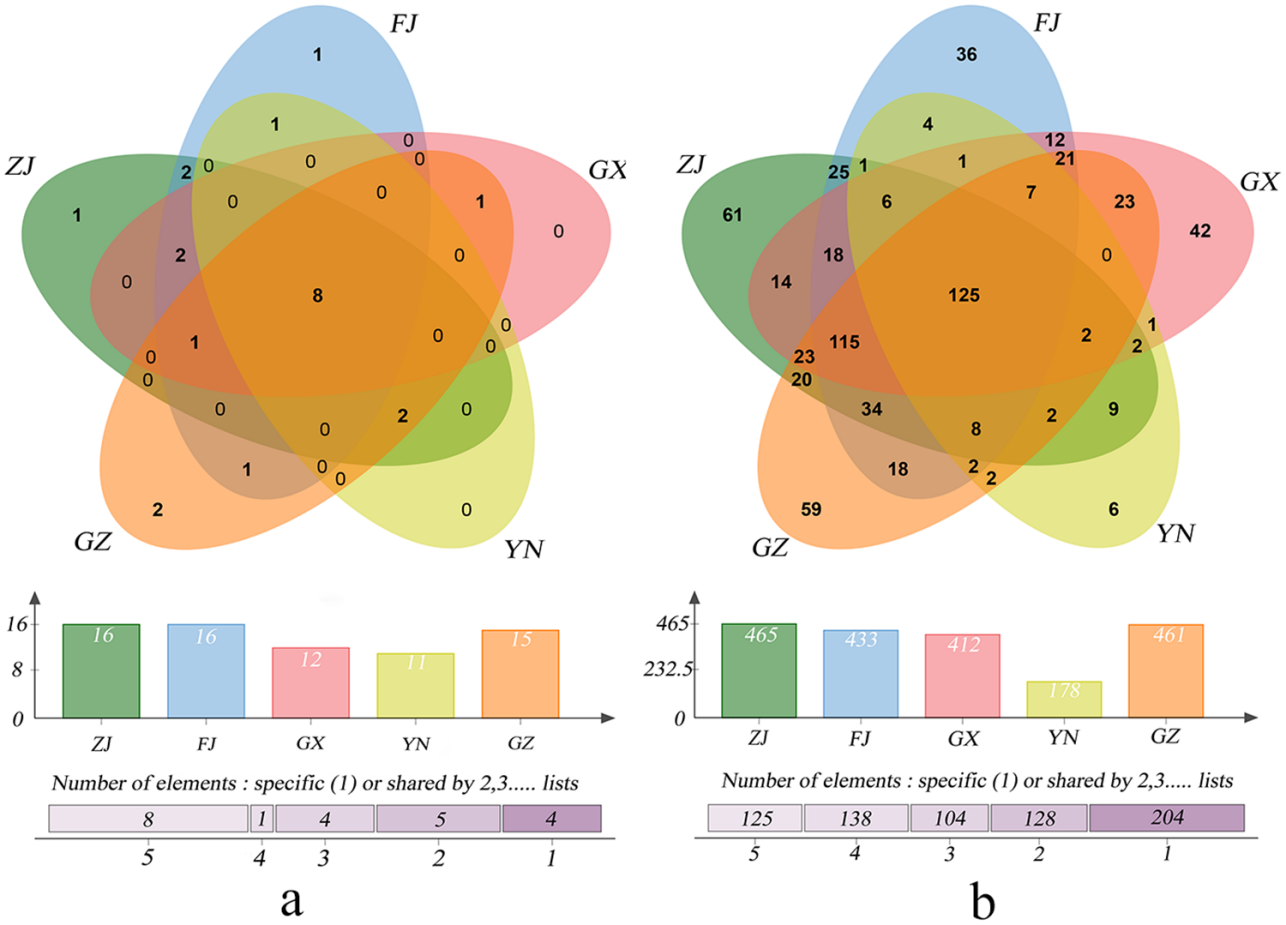
The phylum levels (A) and genus levels (B) of species composition of five planting bases samples.

To investigate the influence of environment on the bacterial communities, the diversity between planting bases was compared. As shown in the Venn diagram (Fig 2), the numbers of phyla were different between bases, with ZJ and FJ having the maximum of 16 phyla, followed by GZ (15), GX (12), and YN (11). Among these phyla, 8 phyla existed in all the planting bases, which occupied 98.94% of the total reads, and 14 phyla were shared by partial bases or unique bases (Fig 2). Interestingly, 4 of the 8 phyla in common dominated in all the bases, and their abundances varied with the planting base. The abundance of *Proteobacteria* varied from 71.81% (GZ) to 96.08% (YN), and that of *Actinobacteria* from 0.36% (YN) to 4.81% (GZ). The relative abundances of *Bacteroidetes* and *Firmicutes* were higher in the GZ samples and lower in the other samples.

On the genus level, some differences existed between the samples of the different planting bases. Firstly, the numbers of genera were different. Sample ZJ had the maximum of 465 genera, followed by GZ (462), FJ (433) and GX (412), and YN had the least number of genera with 179, which was similar to the order of the latitudes of the sampling districts (Table 1). Secondly, the types of genera of each base were different. Except for 125 genera shared by all the planting bases, there were 575 genera shared by partial bases, e.g. 203 genera for 1 base, 129 by 2 bases, 104 and 138 by three and four planting bases respectively. The 203 special genera were unevenly distributed in the different bases, with 61 genera in ZJ, 59 in GZ, 42 in GX, 35 in FJ and 6 in YN, respectively. Thirdly, the abundances of genera in common were unevenly distributed in the different planting bases. Taking the 25 dominant genera with more than 1% reads in at least one base as an example, *Delftia* (10.39–38.42%), *Burkholderia* (2.71–15.98%), *Escherichia/Shigella* (4.90–25.12%), *Pseudomonas* (2.68–30.72%) and *Sphingomonas* (1.83–2.05%) dominated in four of the planting bases except for YN. For the YN sample, the dominant genera only included *Pseudomonas* (57.45%) and *Delftia* (2.72%). In addition, each base had its own dominant genera. There were 7 genera that dominated in the YN sample, e.g. *Aeromonas* 4.82%, and only 1–3 genera in each of the other four bases, with the prominence of *Pantoea* (9.29%) and *Buttiauxella* (6.58%) in GX, and *Methylobacterium* (17.02%) in GZ (Table 3). Overall, the YN sample exhibited unique bacterial composition, while the other four sample locations shared great similarities as well as some differences in their bacterial compositions.

**Table 3:** Bacterial genera with relative abundance >1%.

Item	Root	Stem	Leaf	ZJ	FJ	GX	YN	GZ
*Pseudomonas*	22.06%	20.19%	17.94%	5.47%	30.27%	10.29%	57.45%	2.68%
*Delftia*	9.65%	17.83%	22.14%	38.42%	16.91%	16.69%	2.72%	10.39%
*Escherichia/Shigella*	11.59%	6.69%	10.05%	5.35%	7.35%	4.90%	0.24%	25.12%
*Burkholderia*	5.87%	11.05%	3.12%	6.43%	7.21%	15.98%	0.76%	2.71%
*Methylobacterium*	0.28%	1.81%	11.22%	0.41%	0.74%	1.06%	0.04%	17.02%
*Pantoea*	1.50%	6.05%	0.51%	0.30%	0.80%	9.29%	2.69%	0.21%
*Sphingomonas*	2.21%	0.79%	1.90%	2.05%	2.00%	1.96%	0.23%	1.83%
*Buttiauxella*	3.60%	0.91%	0.25%	0.09%	0.31%	6.58%	0.35%	0.38%
*Duganella*	3.65%	0.16%	0.61%	0.17%	0.79%	0.08%	6.88%	0.12%
*Afipia*	0.56%	0.77%	2.30%	3.53%	0.89%	0.88%	0.07%	0.92%
*Salinarimonas*	0.03%	0.02%	3.23%	0.04%	3.09%	2.42%	0.01%	0.05%
*Acinetobacter*	1.54%	0.69%	0.92%	1.43%	0.43%	0.49%	2.68%	0.52%
*Aeromonas*	0.10%	2.74%	0.11%	0.05%	0.14%	0.11%	4.82%	0.14%
*Pectobacterium*	1.83%	0.12%	0.19%	0.17%	0.07%	0.04%	3.49%	0.12%
*Enterobacter*	0.34%	2.02%	0.09%	0.03%	1.69%	1.70%	0.47%	0.12%
*Yersinia*	0.16%	1.89%	0.11%	0.52%	2.86%	0.02%	0.22%	0.04%
*Streptococcus*	0.01%	1.80%	0.04%	0.07%	0.06%	0.01%	0.004%	2.42%
*Serratia*	0.70%	0.72%	1.23%	0.39%	0.35%	0.35%	1.43%	1.77%
*Ornithobacterium*	0.44%	0.71%	1.05%	0.48%	0.41%	1.30%	0.05%	1.24%
*Nocardioides*	0.17%	0.05%	1.69%	0.18%	0.15%	0.05%	0	2.41%
*Rhodanobacter*	0.64%	0.25%	0.13%	1.14%	0.17%	0.41%	0.05%	0.04%
*Bradyrhizobium*	0.98%	0.10%	0.25%	1.12%	0.74%	0.18%	0.02%	0.24%
*Erwinia*	0.61%	0.55%	0.10%	1.03%	0.09%	0.05%	1.05%	0.06%
*Citrobacter*	0.70%	0.33%	0.34%	0.04%	0.51%	1.54%	0.06%	0.09%
*Klebsiella*	0.37%	0.63%	0.05%	0.44%	0.09%	0.22%	1.02%	0.09%
*Comamonas*	0.60%	0.13%	0.19%	0.19%	0.17%	0.04%	1.21%	0.06%
*Stenotrophomonas*	0.33%	0.77%	0.15%	0.06%	0.09%	0.25%	1.63%	0.17%
*Chryseobacterium*	0.57%	0.25%	0.05%	0.03%	0.03%	0.02%	1.42%	0.06%
*Flavobacterium*	0.40%	0.16%	0.03%	0.02%	0.03%	0.01%	1.01%	0.01%

#### Diversity between tissue types

Interestingly, more phyla were identified in leaves (20) than in stems (16) or in roots (13). Among these phyla, 10 phyla were shared by all the tissues, while 7 were shared by two tissues and 5 existed in unique tissues. Of the 10 phyla in common, 4 were dominant, such as: *Proteobacteria*, *Bacteroidetes*, *Firmicutes*, and *Actinobacteria*, which constituted over 98% of the total reads (S4 Fig). *Proteobacteria* was the most dominant, with 82–88% of the reads in all the tissues; however, the second-most dominant genera was *Bacteroidetes* in the root (4.83%) and leaf (7.12%) samples, and *Firmicutes* was the third, with 7.61% reads in the stem. *Lentisphaerae*, *OD1*, *Deinococcus-Thermus* and *Fibrobacteres* existed only in the root and stem samples, *Spirochaetes* was present only in the roots and stems, and *Chlamydiae* and *Armatimonadetes* were found only in root and leaf samples.

On the genus level, the dominant genera exhibited much greater variability with tissue type. There were 495–506 genera in each tissue, 319 of which existed in all the tissues, while there were 45–61 in every two tissue types, and 70–78 in unique tissues. The 319 genera in common, which constituted 45.64% of the total reads, comprised the remainder of all the genera with over 0.5% reads. There were 10, 10 and 11 genera with over 1% reads in the root, stem, and leaf tissues, respectively. The dominant genera *Pseudomonas* (17.94–22.06%), *Escherichia/Shigella* (6.69–11.59%), *Delftia* (9.65–22.14%) and *Burkholderia* (3.12–11.05%) also dominated in all the tissues, with some variation between tissues. The other dominant genera usually dominated in some unique tissues, e.g. *Methylobacterium* (11.22%) dominated in the leaf, *Pantoea* (6.05%) in the stem, while *Duganella* (3.65%) and *Buttiauxella* (3.60%) were dominant in the root tissues (Table 3). These dominant genera contained most of the bacteria in each tissue, with 65.41%, 67.56% and 70.25% of the reads in the root, stem and leaf samples, respectively. The proportion of abundant and rare species ranged from 48.60% to 60.86% and 39.14% to 51.40% respectively in the *D*. *catenatum* samples (S5 Fig).

### Functional diversity of diazotrophic endophytes

**Fig 3:**
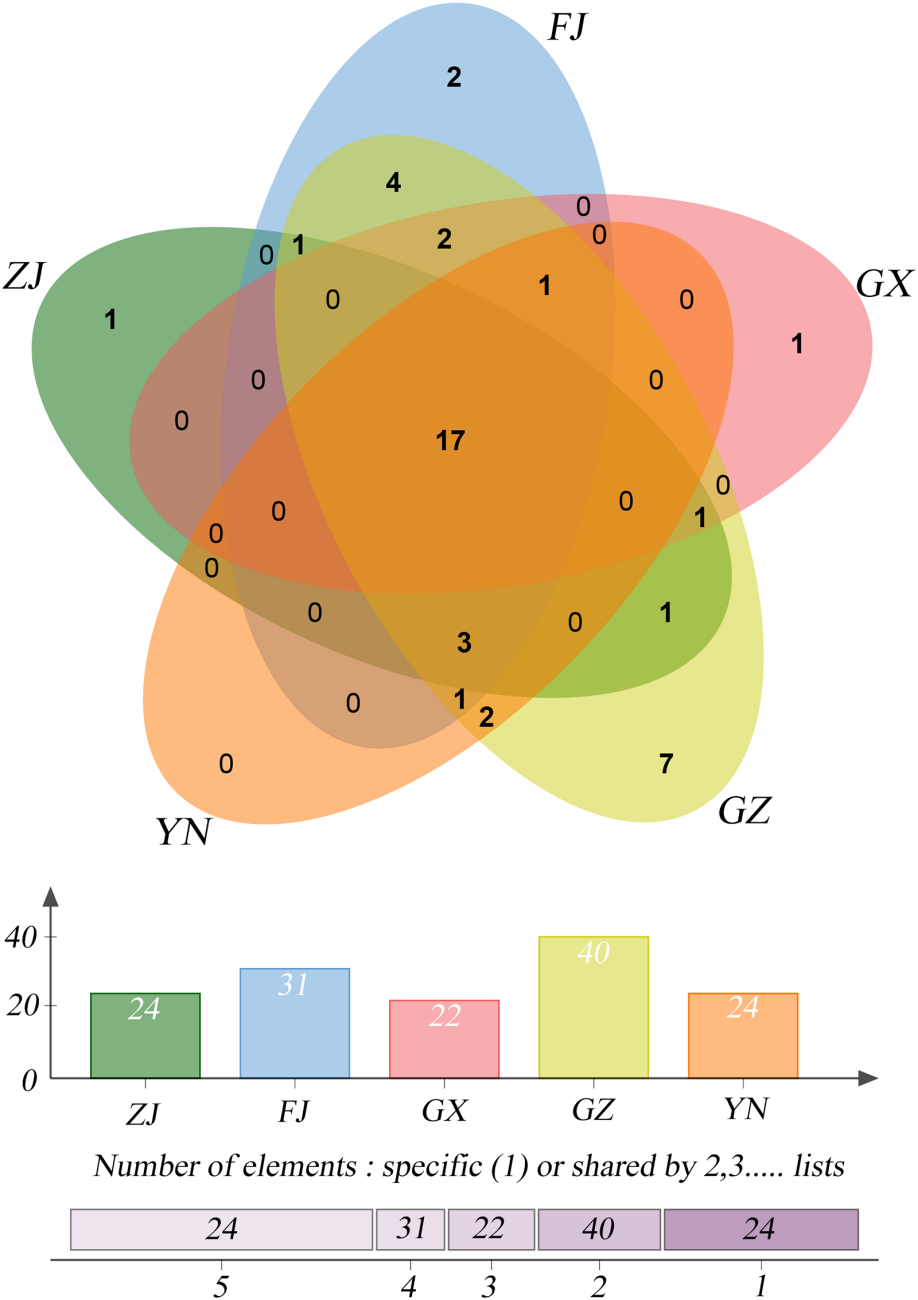
The genus level (meta-*nif*H) of species composition of samples from five planting bases.

To investigate the diversity of diazotrophs in *D*. ***catenatum***, metagenome analysis based on the *nif*H gene was carried out. The rarefaction curves (S1B Fig) all approach a plateau, indicating that further sequencing would not result in more OTUs in each sample. As shown in S2 Table, a total of 4 diazotroph phyla were identified, and *Proteobacteria* was the absolute dominant phylum, with *Alphaproteobacteria* (61.47%) and *Betaproteobacteria* (22.47%) as the leading classes. On the genus level, 45 genera were altogether identified, with 13 genera with over 1% reads constituting 91.75% of the total, while the remaining 32 genera constituted only 1.98% of the total. Among these dominant genera, most of them belonged to *Alphaproteobacteria*, such as: *Bradyrhizobium* (14.85%), *Methylovirgula* (10.68%), *Xanthobacter* (8.91%), *Sphingomonas* (7.13%), *Azospirillum* (5.53%), *Mesorhizobium* (5.45%), *Methylocystis* (4.56%), and *Beijerinckia* (3.74%), which constituted 60.85% of the total reads. In addition, 3 genera, namely, *Burkholderia* (13.19%), *Azohydromonas* (6.12%), and *Herbaspirillum* (2.83%), were divided into *Betaproteobacteria*, which constituted 22.14% of the total reads. The other two genera, *Micrococcus* (6.46%) and *Enterobacter* (2.30%), belonged to *Actinobacteria* and *Gammaproteobacteria*, respectively (Fig 3). According to these results, the potential for nitrogen fixation within the bacterial community is substantial.

#### Diversity indices between planting bases

To determine the effect of environment on the nitrogen-fixers, the diversity between planting bases was compared. As shown in S2 Table, the predominant phylum *Proteobacteria* accounted for a higher percentage (98.99%) in the samples of ZJ, GX and GZ, and a lower percentage in both FJ (66.14%) and YN (69.84%). For the latter two samples, 33.84% reads belonged to unidentified phyla and 30.00% reads belonged to *Actinobacteria*, respectively. Interestingly, *Firmicutes* was observed only in the ZJ, GX, and YN samples with low ratios.

On the genus level, some differences existed between the samples from the different planting bases. Firstly, the numbers of genera were different. Sample YN had the maximum of 40 genera, followed by FJ (33), ZJ and GZ (25), and GX had the least number, with 22 genera. Secondly, the types of genera contained by each base were different. Except for 17 genera shared by all the planting bases, there were 28 genera shared by partial bases, although 26 of those belonged to rare genera with less than 1% of the reads. There were 11 genera for 1 base, 7 for 2 bases, and 5 for both 3 and 4 planting bases, respectively. The 11 special genera were unevenly distributed in different bases, with 6 genera in YN, 3 in FJ, and 1 in both ZJ and GX, respectively. Thirdly, the ratios of the 17 common genera were unevenly distributed in different planting bases (Fig 3). The dominant *Burkholderia* and *Bradyrhizobium* were the most popular genera in four planting bases, followed by *Methylovirgula* and *Mesorhizobium*. Four genera (*Beijerinckia*, *Xanthobacter*, *Sphingomonas* and *Azohydromonas*) dominated in two planting bases, and the other four (*Micrococcus*, *Azospirillum*, *Methylocystis*, and *Herbaspirillum*) dominated in unique bases.

#### Diversity indices between tissue types

Comparing the results between different tissues, some differences were observed on the phylum level. In the root samples, the dominant phyla were: *Proteobacteria* (95.18%), *Actinobacteria* (4.68%), and *Firmicutes* (0.09%). The stem samples had the same phyla with different ratios, with much less *Proteobacteria* (69.46%) and more *Actinobacteria* (13.72%). However, *Proteobacteria* absolutely dominated in the leaves (99.39%), with few *Actinobacteria* (0.04%).

**Fig 4:**
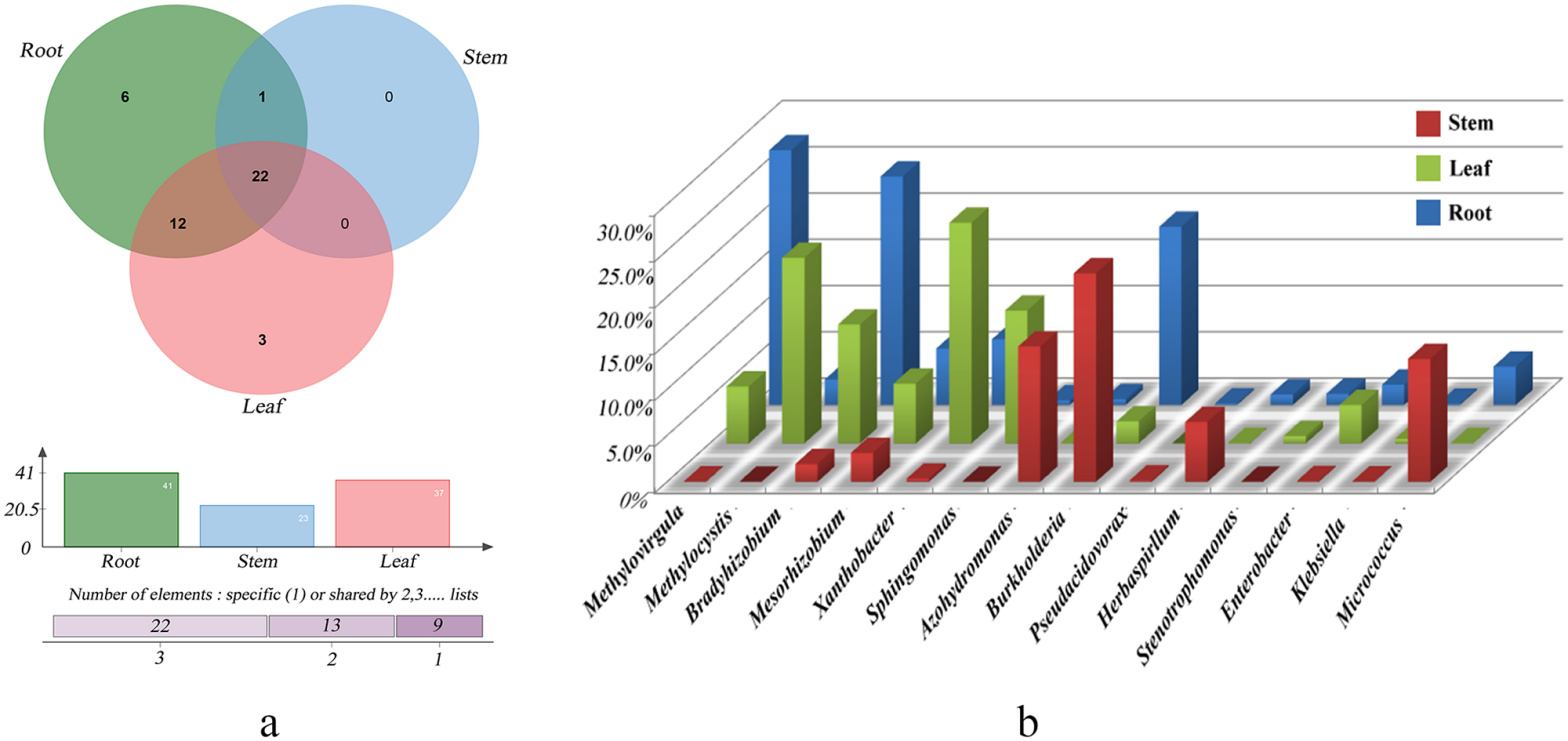
Venn diagram (A) and bar chart (B) for the genus levels of species composition of three tissue samples.

On the genus level, some similarity existed between the samples. Firstly, the numbers of genera were similar between the samples, with 42 for root, 25 for stem and 38 for leaf samples. Secondly, the types of genera were similar among the different tissues. Altogether, there were 24 genera shared by all the tissues, with the only exception of 12 genera shared by root-stem and root-leaf samples (Fig 4), respectively. In the case of the roots, there were nine genera with over 1% of the reads, including six dominant genera, which were *Methylovirgula* (26.85%), *Bradyrhizobium* (28.46%), *Burkholderia* (16.77%), *Mesorhizobium* (7.07%), *Xanthobacter* (5.19%), and *Micrococcus* (4.68%). There were eight genera with over 1% of the reads for the stems, including six dominant genera, which were *Burkholderia* (19.41%), *Azohydromonas* (16.40%), *Azospirillum* (14.74%), *Micrococcus* (13.73%), *Herbaspirillum* (6.69%), and *Beijerinckia* (4.81%). For the leaves, there were ten genera with over 1% reads. The dominant genera were *Xanthobacter* (22.21%), *Sphingomonas* (21.75%), *Bradyrhizobium* (15.35%), *Methylocystis* (11.70%), *Methylovirgula* (6.50%), *Beijerinckia* (5.99%), and *Mesorhizobium* (5.35%) (Fig 4). It is evident that, with the exception of *Mesorhizobium* which dominated in all the tissues, and *Beijerinckia*, *Xanthobacter*, and *Burkholderia* dominating in two types of tissues, the other 9 genera dominated in unique tissues. Thus, the results indicate that there exists high similarity regarding genera type, with some differences in terms of the genera abundances.

### Comparison between *nif*H and 16S rDNA results

**Fig 5:**
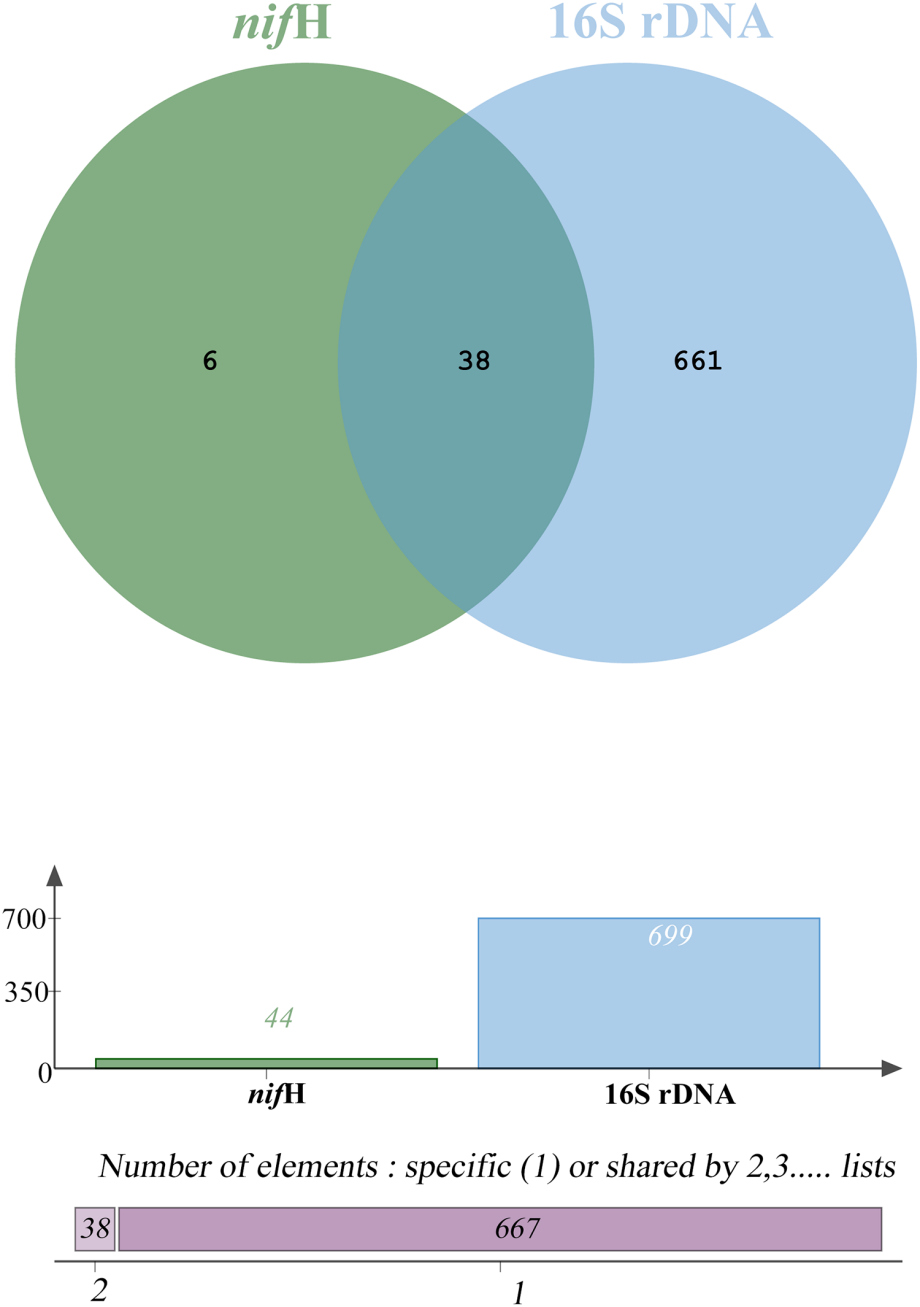
Venn diagram of genus level of species diversity comparison between 16S rDNA and *nif*H methods.

To compare the results between 16S rDNA and *nif*H methods, a Venn diagram was constructed to show their relationship. As shown in Fig 5, 39 out of 45 genera identified from meta-*nif*H were also contained in the results based on meta-16S rRNA. However, six genera, including *Azohydromonas* (6.12%), *Azorhizobium* (0.12%), *Roseiflexus* (0.0015%), *Rhodovulum* (0.0003%), *Halorhodospira* (0.0008%), and *Pelobacter* (0.0002%), were unexpectedly excluded from the meta-16S rDNA results. When comparing the abundances of given genera, only 2 genera (*Burkholderia* and *Sphingomonas*) belong to the dominant bacteria with over 1% ratio in the results obtained using both methods (S3 Table). Some dominant genera from meta-*nif*H, e.g. *Methylovirgula*, *Bradyrhizobium*, *Methylocystis* and *Mesorhizobium*, displayed low ratios in the meta-16S rDNA results. Three dominant genera, including *Azohydromonas*, *Xanthobacter* and *Micrococcus*, were not detected through meta-rDNA. Overall, these observations indicate good consistency in nitrogenase gene diversity and microbial community structure from the cross-system comparison.

## Discussion

It is well known that transplants of different species are difficult to culture without bacteria, hinting at the important ecological roles that bacteria play in plant growth [32]. As epiphytes or lithophytes, orchids principally rely on symbiotic microorganisms for their requisite nutrients. Recently, attention has been paid to the roles of certain bacteria as “satellite” bacteria or mycorrhiza helper bacteria for the promotion of both ectomycorrhiza and the host plant [33]. The roles of orchid-associated bacteria reveal a novel field of symbiosis between microorganisms and orchids.

In this study, the bacterial and diazotrophic diversities in *D*. *catenatum* were successfully characterized based on 16S rRNA and *nif*H genes. Altogether 699 bacterial genera within 22 phyla and 45 diazotrophic genera belonging to 4 phyla were identified from *D*. *catenatum*. The results showed that bacterial and diazotrophic diversities of endophytic bacteria varied with both the tissue types and habitats of *D*. *catenatum*, which needs further evidence.

### Diversity of bacteria in *D*. *catenatum*

Based on the meta-16S rRNA, 22 bacterial phyla were in total identified; 12–17 of these which were distributed in each of the planting bases, and 13–20 in each tissue type. On the genus level, there were in total 699 genera identified from *D*. *catenatum*; 125 of these genera were shared by all the planting bases, and 319 existed in all the tissue types. The occurrence of endophytic bacteria is the result of a combination of several chance factors, determined by the chances of developing roots coming into contact with effective levels of bacteria that can become endophytic, and deterministic factors, determined by the presence of dedicated genetic systems that enable bacterial-plant crosstalk and an active endophytic colonization process [34].

Genetics are a vital factor shaping an endophytic community [35]. In the case of *D*. *catenatum*, within the predominant *Proteobacteria* (71.81–96.08%), *Delftia* (10.39–38.42%), *Pseudomonas* (2.68–30.72%), *Burkholderia* (2.71–15.98%), *Escherichia/Shigella* (4.90–25.12%) and *Sphingomonas* (1.83–2.05%) dominated in four of the planting bases with the exception of YN, which was dominated by *Pseudomonas* (57.45%), and *Delftia* (2.72%). In fact, *Pseudomonas*, *Burkholderia* and *Sphingomonas* are widely distributed in other *Dendrobium* plants, *Orchidaceae* and other habitat environments. For example, *Pseudomonas* was reported to exist in dendrobium plants, such as *Dendrobium moschatum* and *Dendrobium crumenatum* [36, 37], and many orchid plants, including epipactis species [38], *Dactylorhiza maculate* [39], *Paphiopedilum appletonianum* [40], *Acampe papillosa* [41] and *Phalaenopsis* spp. [42]. In summary, *Pseudomonas* is ubiquitous in plants, playing beneficial roles through participation in the carbon and nitrogen cycles and phosphate solubilization [43, 44]. Similar distributions and roles can be found for *Burkholderia* and *Sphingomonas*, as mentioned in the following section.

*Delftia* and *Escherichia/Shigella* have considerable abundances only in *D*. *catenatum*. Usually, the two genera belong to opportunistic pathogens widespread in many habitats, e.g. river [45], soil [46], human gut [47] and plant (*Dendrobium aurantiacum*) [48, 49]. In recent years, *Shigella* was identified as a PGPR [50], and *Delftia* has been found to promote plant growth through suppressing fungal phytopathogens [51], and transforming or degrading multiple organic and inorganic toxins [52, 53]. Regardless of the fact that they are abundant bacteria in *D*. *catenatum* but less so in other plants, it is necessary to test the safety and functions of *Delftia* and *Escherichia/Shigella* in medicinal plants, which are usually freshly eaten.

The environment is another important factor influencing the bacterial communities. In this study, some genera showed plantinging base-specificity (3.53% *Afipia* in ZJ, 3.49% *Pectobacterium* in YN and 2.42% *Streptococcus* in GZ), some displayed tissue-type specificity (3.23% *Salinarimonas* in the leaves, 2.02% *Enterobacter* in the stem), while others showed specificity or dominance in certain tissues from some locations (for example, 17.02% *Methylobacterium* in GZL, 9.29% *Pantoea* in GXS, 6.58% *Buttiauxella* in GXR, 6.88% *Duganella* in YNR and 4.82% *Aeromonas* in YNS). Members of *Methylobacterium* are ubiquitous in leaves, such as those of *Platanus orientalis*, maize, cotton, sunflower, soybean, clover, winter wheat and rice [54–57]. Until now, although no community member of the *Methylobacterium* population was found to colonize only one plant species exclusively [58, 59], the most abundant genera in the leaves of the present study were *Methylobacterium*, consisting of the many species such as *M*. *radiotolerans*, *M*. *mesophilicum*, and *M*. *fujisawaense* [60, 61]. *Pantoea* not only was detected in the stem of *D*. *catenatum*, but also in the stem of sweet potato and willow [62]. While *Buttiauxella* was rarely isolated from roots in previous reports [63], it is possible that the *Buttiauxella* exists specifically in *D*. *catenatum* roots. The base or tissue-specific bacteria displayed the effect of environment on microbial communities. The determination of which resources and functions are important environmental factors requires further study.

In addition to the apparent conspicuous regional characteristics of endophytic bacteria, we also found an interesting phenomenon whereby compared to abundant species, the percentage of rare species determined is also relatively and unusually large, even significantly higher than the abundant species in the YN and GZ samples (S4 Fig). It has long been a belief that abundant species that are represented by perhaps only a few species in the bacterial population play an important role in biotic and abiotic environments. However, the present report provides a testament that rare species also play an important role in biogeochemical cycles and other functional roles in the environment [64]. In addition, the rare species which are unique to a particular sample may play important roles other than those previously predicted. In view of the special efficacy of *D*. *catenatum*, further research regarding the importance of such rare endogenous species is needed to fully understand their role in *D*. *catenatum*.

### Diversity of diazotrophs in *D*. *catenatum*

The abundant bacteria may play a vital role in the growth of the plants. As epiphytic or lithophytic *Dendrobium*, nitrogen fixation might be one of the most important functions of these bacteria in *D*. *catenatum*. Expectedly, 4 phyla and 45 genera of diazotrophs were identified, 17 of which were shared by all the planting bases and 24 shared by all the tissue types. Among the planting bases, *Burkholderia* and *Bradyrhizobium* were the most popular, followed by *Mesorhizobium* and *Methylovirgula*, and *Mesorhizobium* was the most popular in the different tissues, followed by *Beijerinckia*, *Xanthobacter*, and *Burkholderia*. These species might be a good alternative source of nitrogen nutrition, especially for the naturally-grown plants.

*Burkholderia* and *Mesorhizobium* were both encountered in high abundance in all planting bases and tissues, suggesting that they might play a leading role in the microecosystem of *D*. *catenatum*. As additional evidence, we obtained many *Burkholderia* strains from *D*. *catenatum*, which exhibited nitrogen-fixation and thus could promote the growth of *D*. *catenatum* seedlings (data not shown). In fact, *Burkholderia* strains are widely distributed in many plants, including maize [65], rice [66], citrus [67], yellow lupine [68], grape [69], banana and pineapple [70], and display nitrogen-fixing activity in plants [71, 72] which grow well in nutrient-poor sites. In the case of *Mesorhizobium*, it has been successfully used worldwide to permit an effective establishment of the nitrogen-fixing symbiosis with leguminous crop plants [73], and Caragana species in China [74]. In addition, *Mesorhizobium* has shown multiple functions for improving plant growth, such as producing IAA [75], and solubilizing phosphate [76]. *Bradyrhizobium* and *Beijerinckia* have also shown similar characteristics on non-legume plants [77].

Other dominant diazotrophs, such as *Sphingomonas*, *Methylovirgula*, and *Xanthobacter*, are widely distributed in other plants. *Sphingomonas* constituted the dominant endophytic group in *D*. *catenatum*, and is also present in other orchids. Nitrogen-fixing abilities have been confirmed in *Sphingomonas* bacteria associated with rice [78] and *Oryza sativa* [79], and the acetylene was transformed into ethylene [80]. We isolated an endophytic *Sphingomonas* SH1 from *D*. *catenatum*, which promoted the growth of *D*. *catenatum* through production of phytohormones and nitrogen fixation [81]. *Methylovirgula* and *Xanthobacter* are found in water, sediment [82], rice rhizosphere, marigold plants and tree leaves [83]. Interestingly, *Methylovirgula* and *Xanthobacter* belong to both diazotrophic and methylotrophic bacteria [84] in different plants [85]. *Methylovirgula* were specialized to utilize methanol as their sole carbon source [86], and the abundance of *Methylovirgula* correlated significantly and negatively with the C/N ratio [87]. *Xanthobacter flavus* grows autotrophically by using the Calvin cycle for the fixation of CO_2_ [88], and *Xanthobacter autotrophicus* has the ability to grow on H_2_/CO_2_, ketones, alcohols, sugars, carboxylic acids, and aliphatic alkenes [89]. Methylotrophy was reported to be associated with lower plants [90], and it became apparent that extra- and/or intra-cellular symbiotic or mutualistic associations may exist between plants and some methylotrophic strains to make them well suited for survival in stressful environments. *Methylovirgula* and *Xanthobacter* reveal a rich distribution of methylotrophic and diazogtrophic bacteria in *D*. *catenatum*, the roles of which require further investigation.

Previously, cyanobacteria were found to be the first to show a high activity of nitrogen fixation on orchids. Tsavkelova first studied the localization of phototrophic microorganisms on the roots of *Dendrobium moschatum*, *Acampe papillosa*, and *Phalaenopsis amabilis* [91]. *Nostoc*, *Anabaena*, and *Calothrix* were distributed on the surface of the *A*. *papillosa* aerial roots, whereas *Nostoc*, *Oscillatoria*, and representatives of the LPP-group (*Lyngbia*, *Phormidium*, and *Plectonema*, incapable of nitrogen fixation) were found on the substrate roots [92]. The functional characteristics and primary role of the orchid cyanobacteria are those of nitrogen fixation and nutrient supply to both the host plant and the “satellite” microorganisms, including mycorrhizae-forming fungi.

Therefore, the diazotrophs found in this study, as well as cyanobacteria, might be vital sources of nutrient nitrogen for the growth of *D*. *catenatum*, providing the possibility for them to live as epiphytics or lithophytics under wild conditions.

### Difference between bacteria and diazotrophs in *D*. *catenatum*

The results of the current study indicate that the two methods were successful for performing genetic and nitrogen-fixing functional diversity analysis. Comparing the results of the two methods, all phyla and 39 genera were completely overlapped with those based on the 16S rRNA gene, which demonstrated the consistence of the taxonomies based on both the 16S rRNA and *nif*H genes. It further demonstrates the successful use of the *nif*H gene as both a taxonomic and functional marker and target gene in the study of microbial communities.

However, 6 diazotroph genera (*Azohydromonas* 6.12%, *Azorhizobium* 0.12%, *Roseiflexus* 0.0015%, *Rhodovulum* 0.0003%, *Halorhodospira* 0.0008%, and *Pelobacter* 0.0002%) were not covered in the results based on the 16S rRNA gene. There are three possible reasons for this omission. The first reason is the selectivity of the primers. As mentioned before [18], primer pairs targeting the 16S rRNA gene are useful for amplifying bacteria with the exceptions of cyanobacteria and archae. Thus, this explains why no cyanobacteria were detected here; however, it does not explain the additional genus based on the *nif*H gene. The second reason might be a failure of the taxonomy of the *nif*H gene due to horizontal transfer [93], although most of the time it can be used successfully to characterize bacteria. It is reported that some strains displayed two different genera on the basis of the rRNA gene and *nif*H gene [94, 95]. The *nif*H gene from horizontal transfer might confuse or enlarge the diversity of bacteria. Some of the above genera might be attributed to horizontal transfer, which needs further evidence to substantiate. Thirdly, the low ratio of diazotrophs, such as those with less than 0.001% ratio, might influence the competitive amplification of the corresponding 16S rRNA gene. This hypothesis is supported by previous studies that indicated that RNA-targeted probing methods cannot detect target species when numbers are <0.01% of all bacteria present [64, 96]. Therefore, the *nif*H gene is a good alternative target gene for investigating the majority of diazotrophs and their community in plants.

In conclusion, the 16S rRNA and *nif*H genes were successfully used to investigate the diversity of bacteria and diazotrophs in *D*. *catenatum*. Abundant bacteria and diazotrophs were identified in different tissues of *D*. *catenatum* from five different planting bases, many of which were found to be common to the various tissues and planting bases. These bacteria might play a significant role in the provision of nitrogen and other nutrients for the growth of *D*. *catenatum*, which needs further experimental evidence.

## Ethical approval

This article does not contain any studies with human participants performed by any of the authors. All the samples obtained from the planting bases permitted by the owner, one of the co-auther. No endangered or protected species were involved in this study.

## Supporting information

S1 Table Reads information of 15 samples. (XLSX)

S2 Table The proportion of phylum level from different bases (*nif*H). (XLSX)

S3 Table Genus level of species diversity comparison between 16S rDNA and *nif*H. (XLSX)

S1 Fig Shannon curves for 16S rDNA (A) and nifH (B) genes. Samples were collected at 5 sites (ZJ, Zhejiang; FJ, Fujian; GX, Guangxi; YN, Yunnan; GZ, Guizhou).
(TIF)

S2 Fig NMDS plot of the *D*. *catenatum* samples. Most of the samples do not cluster together indicating that the bacterial diversity among the samples is varied and distinct.
(TIF)

S3 Fig Relative abundance of bacterial phyla across the samples. The percentage of sequences is plotted on the Y-axis. Proteobacteria is the predominant phylum in all the samples.
(TIF)

S4 Fig The phylum (A) and genus (B) level of bacterial community composition of the three tissues. (TIF)

S5 Fig Percentage of abundant and rare species across the samples. In all the five sites when compared to abundant species, the percentage of rare species is significantly high indicating the importance of rare species in a community.
(TIF)
