# Supplementary figures and images for: Bacterial and diazotrophic diversities of endophytes in Dendrobium catenatum determined through barcoded pyrosequencing
Source: PLoS One. 2017 Sep 20;12(9):e0184717. doi: 10.1371/journal.pone.0184717 (PMC5607135; doi:10.1371/journal.pone.0184717)

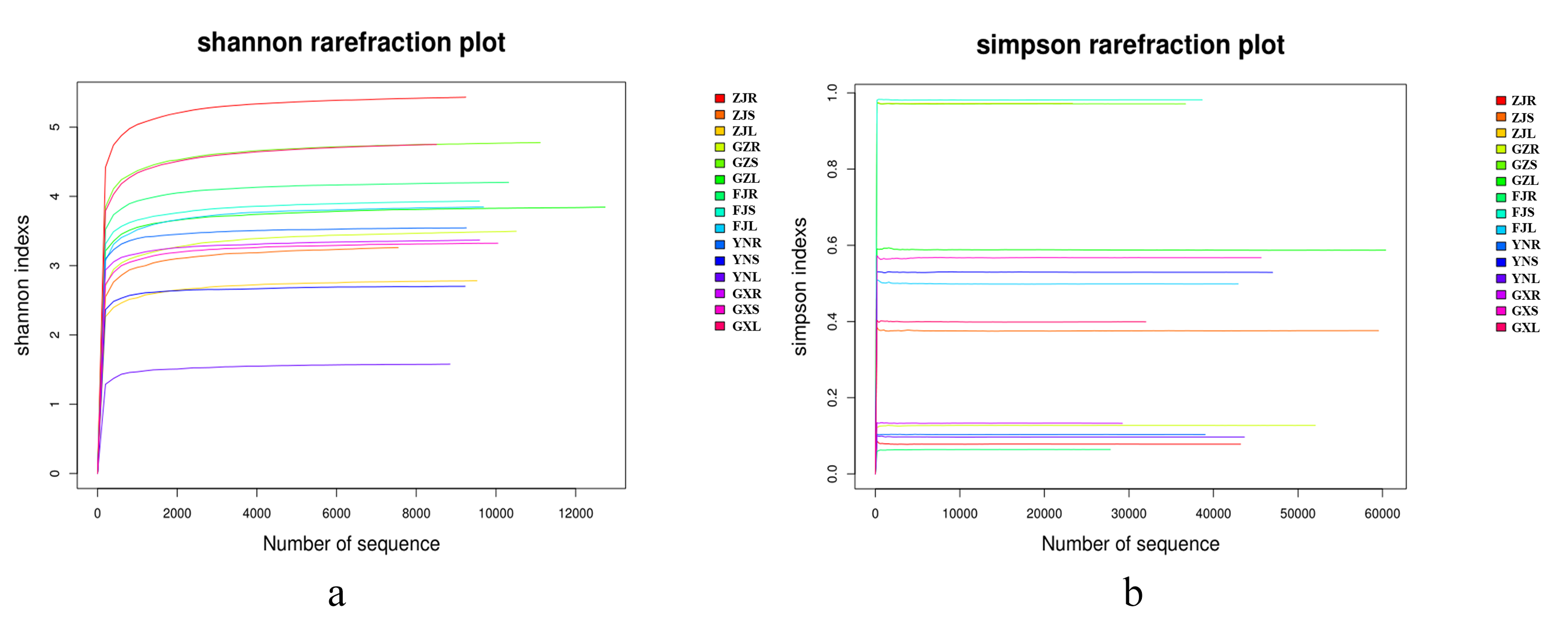

Supplement: S1 Fig — Samples were collected at 5 sites (ZJ, Zhejiang; FJ, Fujian; GX, Guangxi; YN, Yunnan; GZ, Guizhou). (TIF) [file pone.0184717.s004.tif]

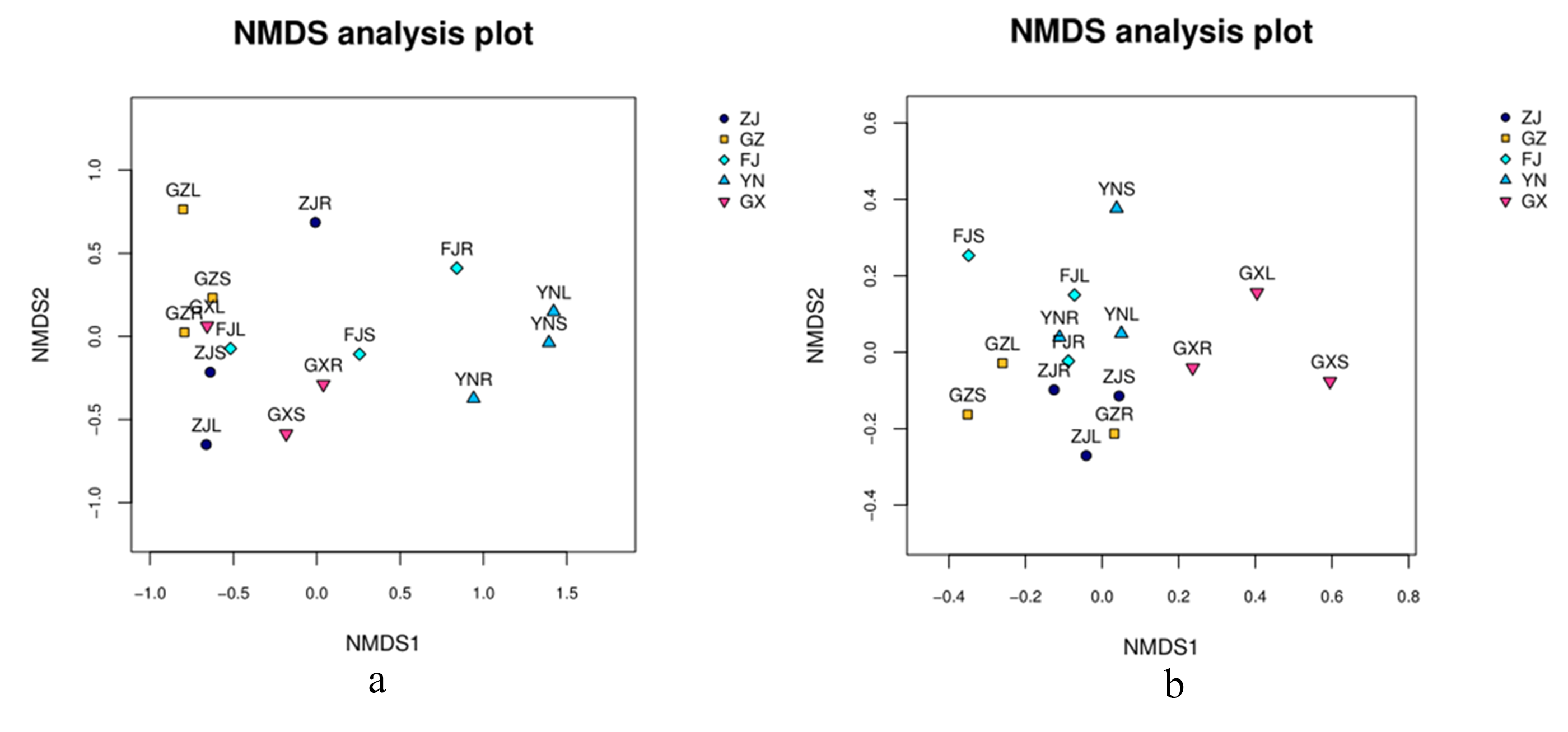

Supplement: S2 Fig — Most of the samples do not cluster together indicating that the bacterial diversity among the samples is varied and distinct. (TIF) [file pone.0184717.s005.tif]

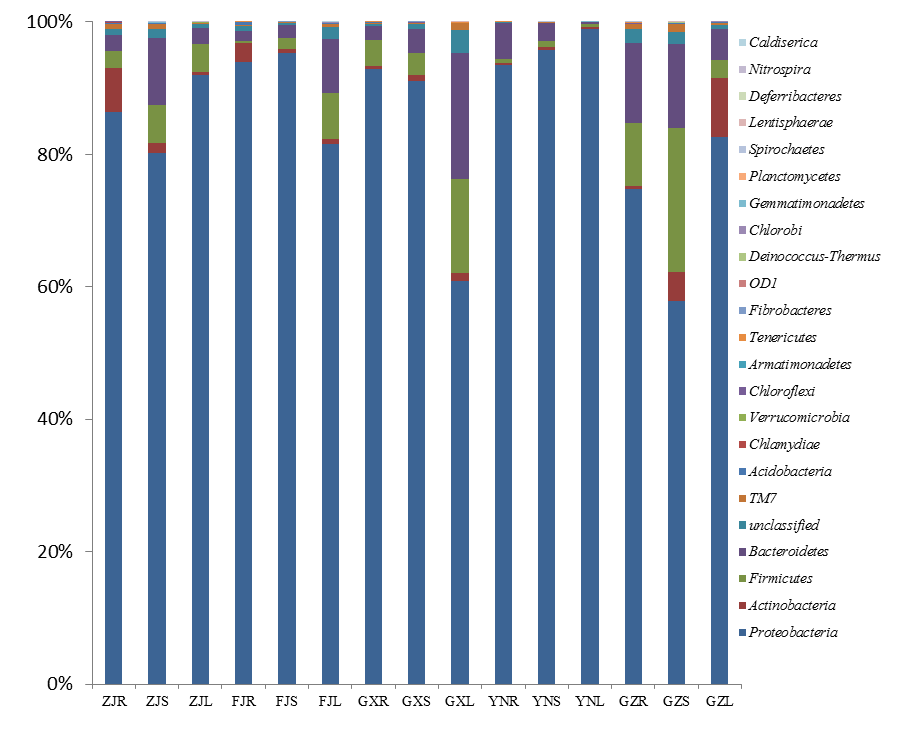

Supplement: S3 Fig — The percentage of sequences is plotted on the Y-axis. Proteobacteria is the predominant phylum in all the samples. (TIF) [file pone.0184717.s006.tif]

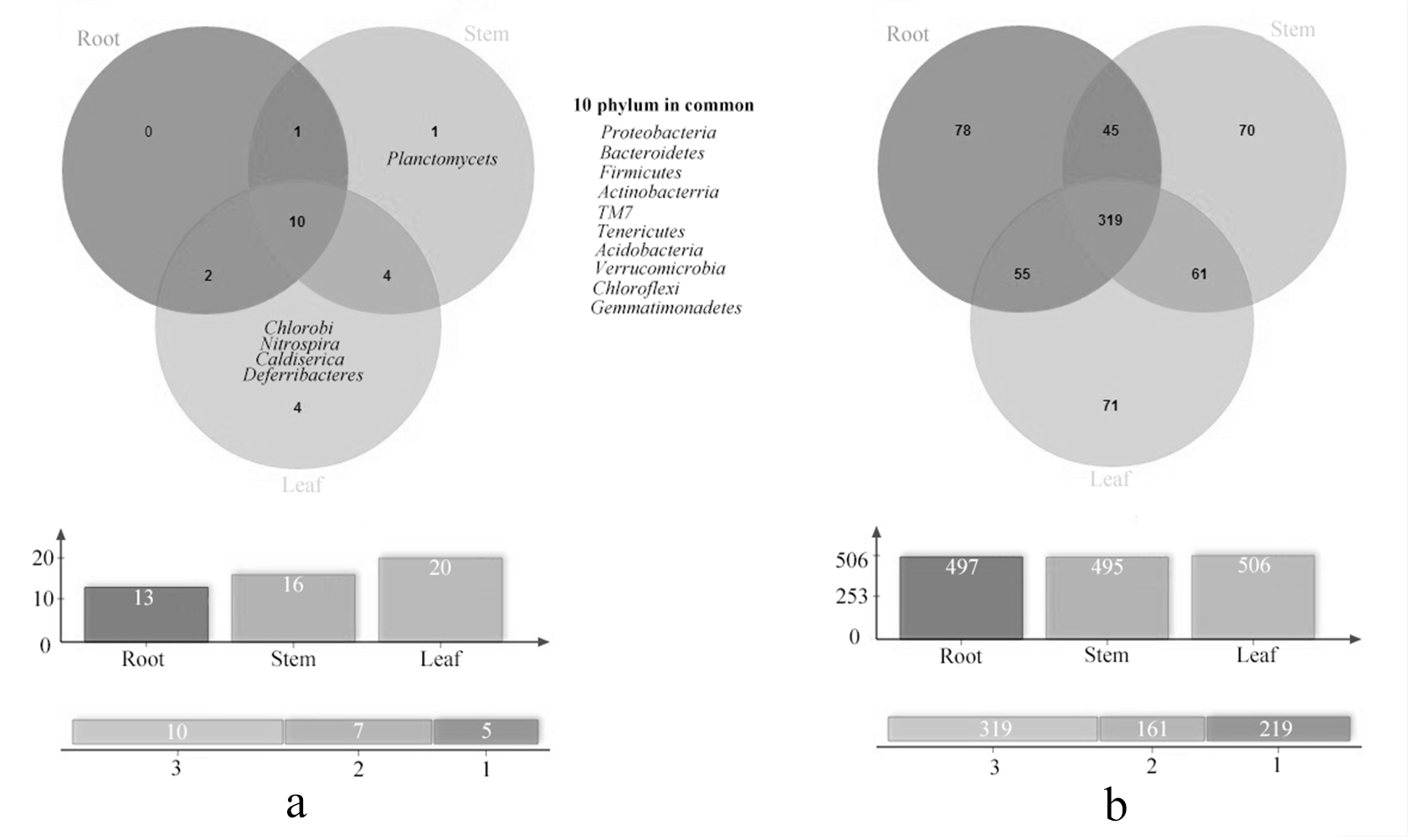

Supplement: S4 Fig — (TIF) [file pone.0184717.s007.tif]

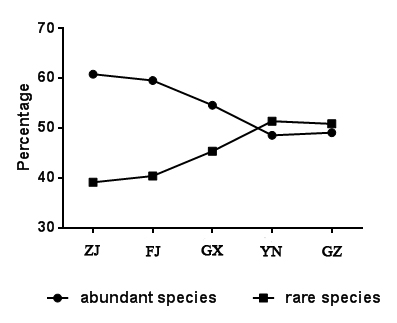

Supplement: S5 Fig — In all the five sites when compared to abundant species, the percentage of rare species is significantly high indicating the importance of rare species in a community. (TIF) [file pone.0184717.s008.tif]
